# An ergonomic study of women workers in a woolen textile factory for identification of health-related problems

**DOI:** 10.4103/0019-5278.40810

**Published:** 2008-04

**Authors:** D. C. Metgud, Subhash Khatri, M. G. Mokashi, P. N. Saha

**Affiliations:** Department of Physiotherapy, KLES Institute of Physiotherapy, Nehru Nagar, Belgaum - 590 010, India

**Keywords:** Ergonomics, health hazards, pain, posture, women workers, woolen industry

## Abstract

The observational cross-sectional study conducted on a sample of 100 women workers who volunteered, outlines their cardio-respiratory and musculo-skeletal profile before, during and at end of work. In addition, information on their health status in general was collected in advance.

Contrary to expectation, there was no significant change in respiratory function. However, the musculo-skeletal problems were found to be abundantly present with pain in 91% of the subjects. Region-wise mapping of pain revealed that postural pain in low back was present in 47% while in neck was 19%. Scapular muscles on the right side were involved in stabilizing shoulder, which never went overhead. On the contrary, left shoulder was raised as high (>90 degrees) in spinning action, while pulling thread. This muscle work involved trapezius, deltoid and triceps action concentrically in lifting and while coming to starting position slowly, eccentrically. There was no pause since the wheel continued to spin the thread continuously, unless a worker opted to stop the work. Accordingly, left wrist and hand were in holding contraction while the right wrist and hand holding the handle were also in a fixed position with wrist in flexion with supinated forearm. Though the overall job was light as per peak HR, there was pain due to fatigue and grip strength weakened by around 10%, at the end of the day's work. In conclusion, pain and fatigue were found to be the main problems for women in the spinning section of the small-scale industry under this study. Women have to take up dual responsibility of a full-time job as well as the domestic work. It was considered that ergonomic factors such as provision of a backrest and frequent rest periods could remediate the musculo-skeletal symptoms.

## INTRODUCTION

Various and manifold is the harvest of diseases reaped by certain workers from the crafts and trades that they pursue; all the profit they get is fatal injury to their health…mostly, from two causes. The first and the most potent is the harmful character of materials that they handle…the second cause I ascribe to certain violent and irregular motions and unnatural postures of the body, by reasons of which the natural structure of the vital machine is so impaired that serious diseases gradually develop therefrom”.[1]

Today is an era of women who have diverse role to play in society. Often they handle two or more tasks simultaneously. They are therefore prone to suffer from work-related diseases, which are further complicated by social, psychological and physiological issues. Roughly, 1 out of 300 female is suffering from some occupation related disease.[2] The working condition of women in India is currently similar to those found in early 19^th^ century in industrial countries.[3]

Since labor intensive economy prevails in India, the musculoskeletal problems may in fact be acute, but insufficient awareness and a lack of proper documentation makes it very difficult to quantify. Through extensive research studies and surveys carried out in different countries, numerous health problems have been identified among industrial workers. But most of the studies were mainly confined to large and medium scale industries in Indian context and less with particular reference to textile industry, excepting the one reported by Kogi from ILO Geneva in small and medium scale industries in developing Asian countries.[4]

However, though ILO's book on occupational health and safety covers woolen textile industry (Hargrave, (D.A,) 1970), the work carried out in this industry is rare, particularly the ergonomic studies in Indian context and with particular reference to women workers.

This factory was located during a visit to the village for clinical teaching in the community-based rehabilitation and it was found out that a number of residents were employed therein. As some women in the spinning section were seen working in the verandah, the spinning movement of the wheel unlike paddling a bicycle, was found to be in the reverse direction, with the initial movement of the upper limb pulling handle towards body. It was therefore considered worthy of studying its ergonomic implications. The woolen industry covered under this study was labor intensive and therefore it was considered necessary to study the health problems. Other than musculo-skeletal problems, there is also a risk of development of chronic respiratory symptoms and impaired lung function.[5] Also studies are rare with respect to respiratory problems in women in spinning section of the woolen industry, hence the need was felt to investigate the respiratory functions in women working in such type of unit.

Ergonomic studies in industrial workers were many, but there were no ergonomic studies in the spinning section of woolen industry. Therefore, it was considered appropriate to take up the issues of ergonomic study of women workers, health and safety aspects in small- scale labor-intensive industry with the following objectives:

1. To identify musculo-skeletal problems among women workers in spinning section of woolen textile industry
2. To identify the risk group pertaining to respiratory disorders
3. To study the volumetric lung functions of the workers with the risk of respiratory disorders
4. To offer suggestions based on ergonomic approach to improve upon work performance

## MATERIALS AND METHODS

Cross-sectional observational type of survey was conducted in spinning section of small scale labor-intensive woolen textile factory by name “Kanakadas Kuri Sangopan Kendra” in village Sindholi of Belgaum district, Karnataka state during the period September 2005 to April 2006. In this study out of 350 workers in the spinning sections, 100 females in the age ranging between 30 to 45 years were randomly selected by convenient sampling from volunteers after taking consent from them. Workers with a background of cardiac, respiratory diseases or accidents affecting musculoskeletal system were excluded from the study.

A pilot study was carried out in various departments of this particular industry and based on the observations made, few problem areas were identified. Among the problems identified, the ergonomically related health problems pertaining to musculo-skeletal and respiratory system in spinning section were selected for the study in consultation with the management. Data were collected using a questionnaire based on workload, working posture and related health and safety problems based on related studies and with suggestions from the experts in the field. The questionnaire consisted of the following items: a) personal details (including sex, age, job tenure, health and medical background); b) musculoskeletal problems in different body regions, [Figure 2 for regions] intensity and duration of pain and whether they were related to posture or activity or both. The site of pain was further assessed by detailed clinical evaluation to know whether it is related to muscle or joint. Respiratory system problems were noted. The questionnaire was filled in by personal interview with the workers. This was followed by recording of radial pulse rate as it is simple, less time-consuming and equally reliable method as energy expenditure advocated by Christensen.[6] Handgrip strength was measured in mid range for the working hand in working position before the women started their work in the morning. Pulse rate was again recorded immediately after they stopped their work for lunch break in the afternoon and then again after the end of day's work in the evening. Handgrip strength was taken at the end of days work. In addition, visual analogue scale which is a straight line with the left end of the line representing no pain and the right end of the line representing the worst pain was used to know the severity of pain at lunch break and at the end of the day's work.[7] Subjects identified with respiratory problems were examined thoroughly and subjected to lung function measurement, using computerized Spirometer.

## JOB DESCRIPTION AND TASK ANALYSIS

The working posture was long-sitting with straight knees and erect back. Legs were parallel with the spinning wheel lengthwise [Figure 3].

Hand was set on the handle at the periphery of the wheel, with forearm in supination and wrist in flexion. Work was carried in the following stages: the movement, if considered with handle of the spinning wheel at the highest point, involved 1) pulling handle back using shoulder extensor-elbow flexor combination of muscle work while midway it changed to shoulder flexor-elbow extensor combination. This combination involved a proximal extensor and flexor work cyclically and in a similar manner at the elbow.

Workers were given 3 kg of raw wool and paid on a fixed rate basis. They could take rest as they needed since there was no fixed rest period other than lunchtime. On an average, they required eight hours to finish the quota of wool allotted to them.

## RESULTS

The data were analyzed as per length of occupational exposure and age of the workers. The number of sites of musculo-skeletal pain were increased with the increase in length of occupational exposure [Table 2].

Table 1 represents the physical characteristics of the workers. Subjects with working tenure between one to five years had maximum pain at 1 and 2 sites constituting 39.4% and subjects with 6-10 years tenure had majority of pain at 1 site constituting 41.9% and subjects with more than 10 years had pain at 2 sites constituting 35.5% of the sample.

**Table 1:** The physical characteristics of the workers

Length of exposure (Yrs)	No. of subject	Age (Yrs)		Ht (Mtrs)		Wt (Kgs)		BMI	
					
		Mean	SD	Mean	SD	Mean	SD	Mean	SD
<1	7	31.80	±03.49	1.47	±0.08	40.40	±03.97	18.66	±00.73
1-5	31	33.73	±03.79	1.46	±0.07	44.15	±05.97	19.86	±02.29
6-10	32	35.06	±04.53	1.49	±0.07	44.11	±05.74	19.62	±02.16
>10	30	37.94	±03.86	1.47	±0.06	44.40	±08.59	20.67	±03.61

**Table 2:** Musculo-skeletal pain based on the number of sites of involvement

Length of exposure (Yrs)	Nil		1 site		2 site		3 site		4 site		5 site		Total
							
	No	%	No	%	No	%	No	%	No	%	No	%	
<1	0	0	2	40.00	2	40	1	20	0	0	0	0	5
1-5	6	18.2	13	39.4	13	39.4	0	0	1	3	0	0	33
6-10	1	3.2	13	41.9	8	25.8	5	16.1	2	6.5	2	6.15	31
>10	2	6.5	7	22.5	11	35.5	8	25.8	3	9.7	0	0	31
Total	9	9	35	35	34	34	14	14	6	6	2	2	100

Table 3 represents the mean visual analogue scale (VAS) scores of the subjects before and after the work. It indicated that there was highly significant association between the pain score and the length of occupational exposure (*P* < .001), except for subjects with less then one year of job exposure.

**Table 3:** VAS score before and after work as per length of occupational exposure

Length of exposure (yrs)	VAS score before work		VAS score after work		Mean increase in VAS score		t	DF	P
						
	Mean	SD	Mean	SD	Mean	SD			
<1	2.70	±01.44	3.10	±01.59	0.4	±0.22	4	4	0.1613^NS^
1-5	2.02	±01.48	2.26	±01.67	0.24	±0.25	5.082	28	0.001^HS^
6-10	2.72	±01.41	2.97	±01.54	0.25	±0.25	5.385	29	0.001^HS^
>10	2.92	±01.84	3.22	±01.98	0.3	±0.24	6.595	29	0.001^HS^

NS - Not significant, HS - Highly significant < .001

**Figure 1:**
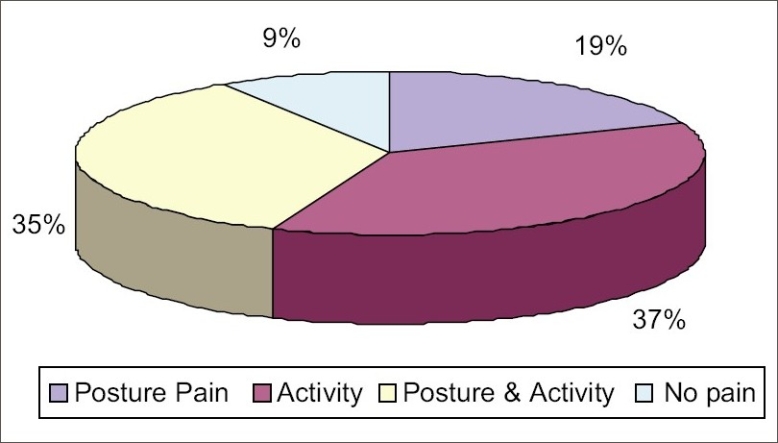
Percentage of posture and activity pain among the workers

Figure 1 indicates that about 35% of the subjects had postural pain that is pain in neck and back due to long sitting for work and also had activity pain in the extremities due to spinning activity. 19% of the subjects had only postural pain and no pain due to spinning activity.

Table 4 shows that there is significant decrease in hand grip strength (*P* < 0.001) with increasing tenure of the work and at the end of the day's work, which indicated muscular fatigue.

**Table 4:** Hand grip strength before and after work as per length of occupational exposure

Length of exposure (yrs)	Hand grip strength (kgs) before work		Hand grip strength (kgs) after work		Mean decrease in grip strength (kgs)		t	DF	P
						
	Mean	SD	Mean	SD	Mean	SD			
<1	9.30	±2.99	8.20	±3.95	1.10	±1.39	1.773	4	0.1581
1-5	10.73	±3.18	9.33	±3.02	1.40	±1.11	7.218	32	0.001^HS^
6-10	9.74	±2.77	8.42	±2.88	1.29	±0.68	10.556	30	0.001^HS^
>10	8.81	±2.94	7.45	±1.35	1.35	±0.62	12.089	30	0.001^HS^

HS = Highly significant < .001

Table 5 shows significant increase in heart rate from beginning of work to lunch break and at the end of day's work with *P* value (*P*<0.001) which also signifies level of fatigue. The rise in heart rate was less than 100 pulses/minute which indicated that the workload in spinning section was light in nature.

**Table 5:** Heart rate responses as per length of occupational exposure

Length of exposure (Yrs)	HR at rest		HR at lunch		HR at end of day		Mean increase In HR at lunch		P	Mean increase in HR at end of day		P
							
	Mean	SD	Mean	SD	Mean	SD	Mean	SD		Mean	SD
<1	69.20	±3.90	77.60	±7.13	83.60	±9.74	8.4	±4.33	0.0123^S^	14.4	±6.61	0.0086^S^
1-5	71.27	±3.16	79.45	±4.59	86.45	±5.34	8.18	±3.14	0.001^HS^	15.18	±4.74	0.001^HS^
6-10	70.58	±3.59	78.13	±4.06	84.06	±5.90	7.55	±3.21	0.001^HS^	13.48	±4.90	0.001^HS^
>10	70.90	±3.38	77.68	±5.01	83.34	±6.01	6.77	±2.77	0.001^HS^	12.94	±4.58	0.001^HS^

S = Significant, HS = Highly significant < .001

Table 6 represents the spirometric findings of the symptomatic and control group which was not statistically significant for FVC, FEV1, PEFR and FEV% indicating that none of the subjects suffered from any kind of respiratory problems.

**Table 6:** Spirometric findings of control and symptomatic group

	Symptomatic group (*n* = 9)			Control group (*n* = 9)	t	DF	P
								
	M	SD	M	SD			
Ht	1.45	±0.05	1.47	±0.05			
Wt	46.17	±8.89	45.33	±5.32	0.24	16	0.81^NS^
BMI	21.47	±2.92	21.08	±1.72	0.34	16	0.73^NS^
FVC	1.80	±0.43	2.02	±0.44	1.07	16	0.29^NS^
FEV1	1.66	±0.42	1.90	±0.38	1.27	16	0.22^NS^
PEFR	3.31	±1.14	3.59	±1.29	0.48	16	0.63^NS^
FEV%	90.22	±6.98	94.67	±6.16	1.43	16	0.17^NS^

NS - Not significant, *n* = No. of subjects

## DISCUSSION

Our study showed that 91% of the subjects suffered from at least one work-related musculo-skeletal pain in relation to length of occupational exposure. Similar study was done by Montrenils, Laflames and Pellier on textile tufting workers handling thread cone as per length of occupational exposure and have reported that 64.9% had one work-related musculo-skeletal pain.[8] This is significant support to our study, however our study differs from this study because in addition to percentage of musculo-skeletal pain we have studied the number of sites of pain due to work and further analyzed whether pain was due to working posture only or spinning activity only or related to both working posture and spinning activity. There was 10-15% rise in pain at the end of day's work as recorded by pain score. The rise in intensity of pain could be due to static work posture, articular wear and tear that may have resulted due to the repetitive nature of the job with occupational exposure of one to five years because more number of subjects that is 33% were in this group. Phekhlinanar had reported that with regard to the length of exposure of three to five years and age of the individual which was an expression of summed effect of articular wear out and chronic micro traumatism in a study on osteodystrophic changes in the hands of female textile workers[9] which is similar to our findings. Majority of the subjects in our study had shoulder and wrist pain which could be due to the repetitive nature of the job (Cumulative Trauma Disorder-CTD) and the poor design of the spinning wheel.[10] Similar findings had been reported by Punnelt, Robins Keyserling in female garment workers.[11]

**Figure 2:**
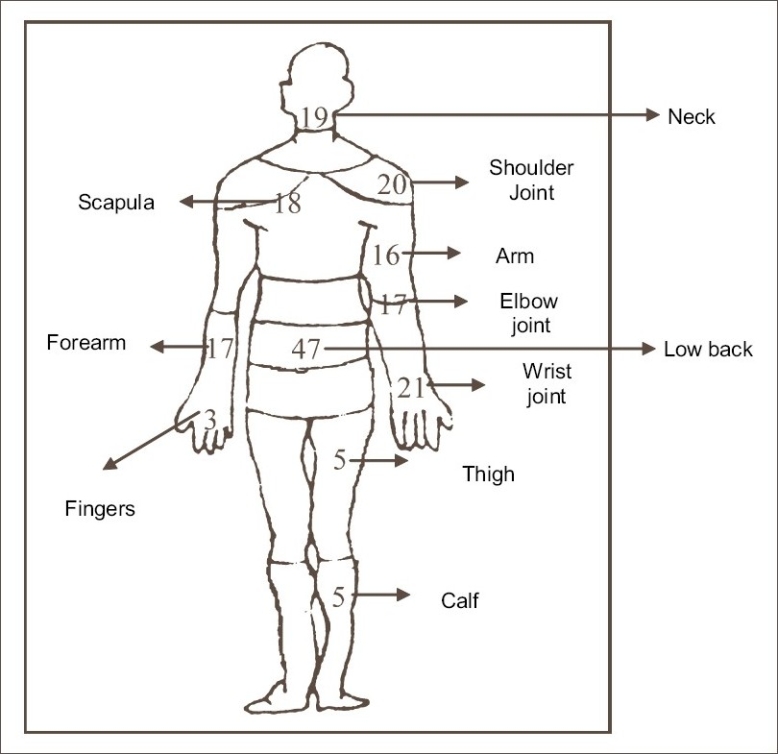
Incidence of musculo-skeletal pain site wise in the women workers

[Figure 2] shows pain in various parts of the body of which subjects with neck pain were 19 and back pain were 47.[12,13]

**Figure 3:**
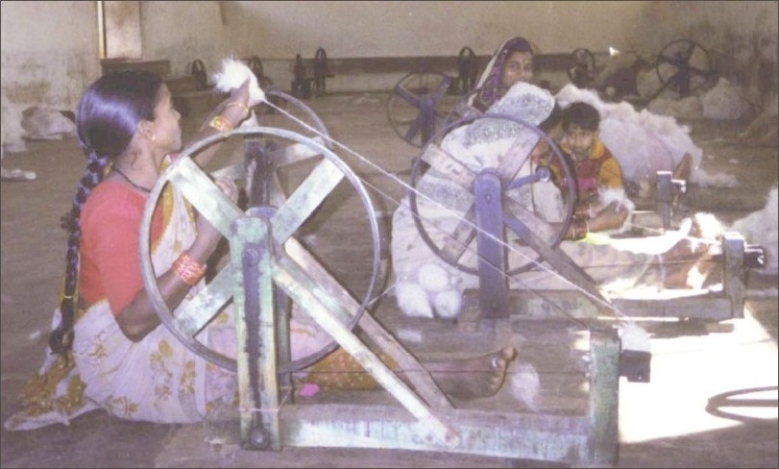
Work posture

This was because the women in our study were in a long sitting posture without backrest and maintained this position for at least eight hours a day. Grand Jean has reported that sitting posture has a disadvantage because it affects digestion and breathing due to prolonged slacking of the abdominal musculature and the purported ill effect of the flexion of the lumbar spine.[14] Since the women in our study were involved in two jobs, spinning and farm work as means of income to support their family and also being married had family responsibilities and household work also which could be a major factor for their residual musculo-skeletal pain. “Women have to assume greater responsibilities, compelling them to take up employment outside the home, while continuing at the same time with their traditional domestic work. Studies by different professional groups revealed that women were exploited without any consideration of workload demands, physical fitness, nutritional status and their biological status as they performed a dual role. As a result, the aggregate workload placed on them was so high that it became incompatible with their physical fitness leading to fatigue thereby lowering efficiency and impairing health in the long run”.[15]

Since health itself is being viewed upon in socio-economic context, the problem of pain and fatigue has multifarious dimensions. Especially the nutritional status needs to be probed. In general, in developing countries, consumption of carbohydrates is more than that of proteins in any form. This has direct bearing on energy metabolism. As there is residual pain and fatigue, the productivity slows down. This compels the workers to speed up the work resulting in increase in symptoms setting up a vicious cycle. Remedy is the rest, which is bypassed in the process of reaching the target. For the spinning hand work is cyclic and both the agonists and the antagonists get some rest through the inhibition of impulses. However, the left upper limb and hand pulling the thread out, has no rest since the same muscles work during pulling up concentrically and while bringing it down eccentrically. Unless design of the Charakha is modified, this cannot be rectified.

The decreased handgrip strength values obtained in the study indicated that all subjects had general muscular fatigue at the end of day's work irrespective of age and length of exposure, which could be caused by repetitive movement of the working hand. Keren and Chand also reported similar changes in hand grip strength.[16] Also small muscles fatigue and wrist pain could contribute to hand pain.

Tomas, had analyzed the dependency between occupational work performance and heart rate.[17] In our study also there was mean increase in pulse rate by15 beats/minute from the beginning of the day's work to end of the day's work. There was a progressive rise in pulse rate with time indicating physiological fatigue. But the maximum heart rate recorded while performing spinning activity was below 100 beats/minute indicating that the workload was light.[6,15] These findings are in accordance with the findings of Varghese *et al*., who reported that the acceptable limits of physiological workload among women workers as determined from energy expenditure and heart rate were found to be 10 kj/min. and 110 bpm respectively for eight-hours continuous work.[18] Saha *et al*., has reported that “acceptable workload” (AWL) should not exceed 35% of an individual's aerobic power (VO2 max) for Indian women workers.[19]

Although the nature of work was light as evaluated by heart rate responses, prolonged sitting in a particular posture might have resulted in fatigue in localized muscles, referred to as “static muscular fatigue”. This might be the reason for the progressive rise in heart rate observed.[20,21] We presume that the ambient temperature was not a variable for rise in heart rate or fatigue at the time of study as the recorded temperature was between 28° and 30°C. (which is a permissible limit for light manual work as per Central Labor Institute, Bombay).

About 9% of the subjects had been identified with respiratory disease with grade 3 dyspnoea, which could be due to exposure to wool and farm dust for a long period.[22] Spirometry was performed on subjects who were identified from the questionnaire as affected with respiratory problems and were compared with the control group matched for age and height and was statistically not significant which indicated that respiratory problems were negligible in the spinning section of the woolen industry. In our study, we had taken parameters like FVC, PEFR and FEV% rather then only FEV1 so that we get a clear picture of the type of respiratory disorder that is whether restrictive or obstructive. The Spiro metric values obtained for both control and symptomatic group showed marked difference by a margin of 20 to 30% from the predicted values, but the predicted values by the apparatus were for western population. So the results couldn't be compared as the predicted values for Indian population differ significantly.

### Suggestions for work modification

1. In order to decrease fatigue it is advisable to provide a frequent short pause that is 10 min followed by every 50 min of work. The rest period should not be spent while sitting in the place of work.
2. Seats with adjustable back rest supporting the lumbar region are recommended to reduce postural strain and low back pain, which is likely to result in the long run without any back support.
3. The axis of the wheel should be at the same height as the axis of the shoulder to avoid extra muscular effort and discomfort/pain to the workers.

**Comments:** The change in the rest period was up to the workers. They want to finish work as early as possible. They were free to work even with staggered timings. Further work was light and did not pose any stress on the cardio-respiratory function, compelling them to take rest. Muscle pain and fatigue was accepted by the women as a part of job rather than a health hazard. Pain was not severe enough to stop the work.

Backrest has to be provided by the factory, in some form. Even if it moderates low back pain, the pattern of pain at other parts of body would continue due to the nature of work itself. Cervical pain and pain in the muscles in the upper limbs could be alleviated by intermittent rest periods, which need to be determined.

It is advisable that the workstation of the spinning section be redesigned in the light of the above recommendations and studied ergonomically, considering human capabilities and limitations from anatomical and physiological viewpoint. The purpose is to ensure health with comfort, safety and well-being of the employees.

## CONCLUSION

Although the workload was light, incidence of musculo-skeletal pain was high (91%), indicating that there were definite ergonomic factors responsible for the musculo-skeletal problems. So based on the observations made in this study it could be concluded that there is ample scope for improvement in work design, machine layout and working conditions in the unit under study from ergonomic view point with the objective of providing maximum comfort to the women workers for promotion of their health and well being and consequently enhancement of productivity.
